# Is ankle taping effective to limit the ankle dorsiflexion in a single-training session? An observational study in semi-professional basketball players

**DOI:** 10.1590/1516-3180.2022.0578.R1.06032023

**Published:** 2023-07-31

**Authors:** Carlos Romero-Morales, Isabel Pedraza-García, Daniel López-López, Luis Berlanga, Blanca de la Cruz, César Calvo-Lobo, Fernando García-Sanz

**Affiliations:** I PhD. Senior Lecturer, Faculty of Sport Sciences, Universidad Europea de Madrid, Madrid, Spain.; II MSc. Lecturer, Faculty of Sport Sciences, Universidad Europea de Madrid, Madrid, Spain.; III MSc, MPH, BSC, PhD, and DPM. Senior Lecturer, Research, Health and Podiatry Group. Department of Health Sciences. Faculty of Nursing and Podiatry. Industrial Campus of Ferrol. Universidade da Coruña, Spain.; IV PhD. Physical Activity and Sports, Centro de Estudios Universitarios Cardenal Spínola CEU, Sevilla, Spain. Exercise Physiology Group, Faculty of Health Sciences, Universidad Francisco de Vitoria, Madrid, Spain.; Universidad Francisco de Vitoria, Faculty of Health Sciences, Madrid, Spain; V MSc, PhD. Senior lecturer, Department of Physiotherapy, University of Seville, Seville, Spain.; VI PT, MSc, PhD. Lecturer, School of Nursing, Physiotherapy and Podiatry, Universidad Complutense de Madrid, Madrid, Spain.; VII MSc. Lecturer, Clinica CEMTRO, Madrid, Spain.

**Keywords:** Foot, Sport, Ankle, Biomechanics, Taping, Prevention, Ankle sprain, Personalized treatment, Range of motion

## Abstract

**BACKGROUND:**

Ankle taping (AT) is effective in preventing ankle sprain injuries in most common sports and is employed in rehabilitation and prevention sports.

**OBJECTIVE:**

This study aimed to investigate the effectiveness of AT to restricting excessive frontal plane ankle movements in semi-professional basketball players throughout the training session.

**DESIGN AND SETTING:**

A cross-sectional study was performed at the Universidad Europea de Madrid.

**METHODS:**

Forty male and female semi-professional basketball players were divided into two groups. The ankle dorsiflexion range of motion (ROM) and interlimb asymmetries in a weight-bearing lunge position were evaluated at four time points: 1) with no tape, 2) before practice, at 30 min of practice, and 3) immediately after practice.

**RESULTS:**

In male basketball players, no differences were observed in the right and left ankles between the baseline and 30 min and between baseline and 90 min of assessment. In female athletes, significant differences were reported between baseline and pre-training assessments for the right ankle and also significant differences between baseline and 90 min in both ankles.

**CONCLUSIONS:**

Ankle taping effectively decreased the ankle dorsiflexion ROM in male and female basketball players immediately after application. However, ROM restriction was very low after 30 and 90 min, as assessed in a single basketball practice. Therefore, the classic taping method should be revised to develop new prophylactic approaches, such as the implementation of semi-rigid bracing techniques or the addition of active stripes during training or game pauses.

## INTRODUCTION

It is known that ankle taping (AT) is effective in preventing ankle sprain injuries in the most common sports (e.g., basketball and soccer). Current research suggests that prophylactic approaches, such as taping or bracing, are effective in protecting the ligaments and soft tissues in maximal stress situations.^1^ AT is employed in the rehabilitation and prevention context, both in sports and non-sports populations. However, players who practice jumping and repeated landings commonly use AT as a prophylactic method to restrict the ankle range of motion (ROM).^2^ Additionally, it is associated with competition or training moments with the aim of reducing the incidence of ankle sprains. Several factors were described in individuals who use AT approaches, for example, Karlsson and Adreasson described a decrease of the peroneus muscle contraction time evaluated with electromyography.^3^ The effectiveness of AT to decrease the average inversion velocity, maximum inversion velocity, and time to maximum inversion velocity have been analyzed; however, no differences were observed between individuals with and without pre-wrap pads.^4^ Regarding the effect of AT in rugby players, taping of the ankle joint was effective in decreasing the inversion ROM.^5^ Moreover, Callaghan et al. showed a limited ankle eversion-inversion ROM in individuals with AT in the non-weight bearing position.^6^ A systematic review developed by Kerkhoffs et al.^7^ supports the fact that the AT and elastic bandage were considered effective to reduce the ankle dorsiflexion ROM. Similarly, Kemler et al.^8^ carried out a systematic review showing the benefits of elastic bandages and AT in individuals with ankle sprain episodes. Taping applications have been widely extended, and several athletes learn the taping technique. For example, Smyth et al. assessed AT with and without self-application and reported the benefits on the proprioception aspect.^9^ However, several authors reported non-desired effects on the lower limb biomechanics and sports tasks. In this context, McCaw et al. and Riemann et al. reported a decrease in the jump performance time to reach forces in the landing phase.^10,11^ Skin disturbances, such as erythema or irritation have also been reported in subjects who have to perform AT repeatedly.^7^

Currently, the ankle sprain has been reported as the most common injury in sports.^12^ This condition shows an incidence ratio of 3.85 per 1,000 participants in basketball players, with the landing phase being the main cause of injury.^13^ Therefore, biomedical staff have focused on over-plantar flexion biomechanics that occur during running or landing considering the ankle joint position as one of the main injuries in basketball players.^14^

Current research reported that AT is effective in preventing and reducing the incidence and severity of ankle sprains in basketball players during practice or games. Romero et al.^15^ in previous research showed that AT was effective in basketball players at the beginning of practice; however, at the end of practice, the taping effect for ROM restriction was very low. Thus, the aim of the present study was to investigate the effectiveness of AT for ankle joint ROM restriction in semi-professional basketball players throughout a training session. Thus, we assessed the ankle dorsiflexion ROM in a weight-bearing lunge position at four time-points: 1) with no tape, 2) before practice, at 30-min after practice, and 3) immediately after practice. Based on previous research and our clinical experience, we hypothesized that taping had lost the initial effectiveness for restricting the ankle ROM in the first 30 min of the training session, substantially decreasing the joint restriction, which was the second part of the session in which there was a high injury risk for basketball players.

## OBJECTIVE

The aim of the present study was to investigate the effectiveness of AT in restricting excessive frontal plane ankle movements in semiprofessional basketball players throughout the training session.

## METHODS

A cross-sectional observational study was conducted in January 2022 following the Strengthening the Reporting of Observational Studies in Epidemiology (STROBE) guidelines.^16^

### Ethical statement

This study was approved by the Research and Ethics Committee of the Universidad Europea de Madrid (CIPI/19/157; December 10, 2021). Before participating in the study, the players were fully informed about the protocol, and written informed consent was obtained from their parents. The Declaration of Helsinki was adhered to throughout this study.

### Participants

A total sample of 40 semi-professional basketball players were enrolled in the present study and divided into two groups: group A composed by 20 male basketball players (20.00±6.00 years) and group B composed by 20 female basketball players. (24.00±3.50 years). Both ankle joints of all players were taped, usually as prescribed by a specialized medical doctor. The players recruited for the study belonged to male and female basketball teams that played in the third Spanish basketball division. Semi-professional individuals followed a training schedule of 2 h per day, 4 days per week, and played one to two matches in a week.^17^ The subjects were excluded if they underwent a physical therapy treatment program, suffered any musculoskeletal condition in the last six weeks, had skin allergies, and any history of lower limb surgery, did not complete all the training sessions, or had other foot orthoses.^15^

### Taping procedure

All taping procedures were developed by the same therapist with two years of experience in sports taping methods^18^ according to the Sport Medicine protocols.^19^ Before the taping procedure, both ankles were covered by a pre-wrap in order to avoid skin disturbances for repetitive daily use.^20^ Following the procedure described by Williams et al., two strips were applied around the leg, 10 cm above the tibialis malleoli with a 38-mm self-adhesive tape with the foot placed in a neutral position.^18^ Subsequently, two strips were placed from the medial to the lateral side of the ankle. Finally, the classic “figure sixes” for the subtalar joint were initially placed onto the medial anchor through the plantar surface of the foot to attach back onto the medial anchor. To complete the AT procedure, all free endings and spaces were not covered with tape. All procedures were performed with a classic rigid tape, employed in all Spanish basketball male and female divisions as a prophylactic method for ankle injury prevention.

### Basketball training sessions

All training sessions comprised a 90 min technical session and were structured into three stages: warm-up (15 min), tactical skills (15 min), and game situations (60 min).

### Outcome measurements

All ankle ROM assessments were performed using the Dorsiflex application (v.2.0; Balsalobre-Fernández, 2017, Madrid, Spain) installed on an iPhone 8 (iOS 12.1; Apple Inc., Cupertino, California, United States). The Dorsiflex application is considered a reliable and valid mobile app for the assessment of the ankle ROM and asymmetries between legs in the weight-bearing position.^21^ To check the ankle ROM, the iPhone was located at the tibial anterior tuberosity to evaluate the ankle between the tibia and the ground in the weight-bearing position. This procedure was developed for each subject for both legs, and the Dorsiflex application also reported an asymmetry index between the legs. The assessments were performed in four periods: 1) baseline, before the practice without bandaging; 2) pre-training, immediately after the baseline measurement; 3) at 30 min of practice; and (4) immediately after the end of the training session.

### Statistics

SPSS v.23 (IBM SPSS Statistics for MacOS, New York, NY, USA) was used for the statistical analysis. First, the Shapiro–Wilk test was used to check the normality assumption. For each group, a one-way analysis of variance (ANOVA) and Bonferroni's correction were employed to assess the significant differences between the four time points (baseline, pre-training, 30 min of training, and post-training) and check for multiple comparisons. In addition, the effect size was calculated using a partial Eta^2^ coefficient.

To evaluate the differences between groups, the Student's *t*-test and Mann–Whitney U test were used for parametric and non-parametric data for the sociodemographic groups, respectively. To evaluate the effects of time and time versus group on the dependent variables, a two-way ANOVA analysis was performed for repeated measures. The Greenhouse–Geisser correction was also applied when the Mauchly test rejected sphericity. In addition, a Bonferroni post–hoc analysis was used for multiple comparisons, and the effect size was calculated using the Eta^2^ coefficient. Thorough the study, the level of significance was set at P < 0.05, with an β error of 0.05, 95% confidence interval [CI]), and the desired power of 80% (β error of 0.2).

## RESULTS

Considering Table 1 and as expected, due to sex characteristics, height and weight differences were reported between the groups (Table 1). In male basketball players, significant differences were observed in the asymmetry variable (f = 5.510; P = 0.002 [0.186]), and no differences were observed between the right and left ankles. In the female group, significant differences were reported for the right [f = 6.925; P = 0.001] and left ankles [f = 5.373, P = 0.002] (Table 2). Post-hoc Bonferroni analyses showed significant differences between the baseline and pre-training assessments for male players and between baseline and 30-min assessments. In addition, for the female group, Bonferroni corrections showed significant differences for the right ankle between pre-training and post-training and between baseline and pre-training evaluations. The left ankle showed significant differences between pre-training and post-training assessments (Figure 2).

**Table 1:** Sociodemographic data of the sample

Data	Men (n = 20)	Women (n = 20)	Total sample (n = 40)	P value
Age, years	20.00 ± 6.00†	24.00 ± 3.50*	24.00 ± 6.00†	0.127††
Height, m	1.87 ± 0.10†	1.72 ± 0.08†	1.80 ± 0.17†	0.001††
Weight, kg	82.00 ± 6.75†	71.25 ± 9.64*	77.00 ± 15.00*	0.001**
BMI (kg/m^2^)	22.61 ± 1.63*	21.93 ± 2.53*	23.80 ± 2.11†	0.569††

* Mean ± standard deviation was applied;

** The Student T-test was performed for independent samples;

† Median ± interquartile range was used;

†† The Mann–Whitney U test was performed.

**Table 2:** One-way analysis of variance for the ankle range of motion and asymmetry variables

Group		Baseline	Pre-training	30-min training	Post-training	Time f; P (Eta^2^)
**Male**						
	Right ankle	39.27 ± 5.49	36.96 ± 5.07	37.68 ± 4.77	40.45 ± 5.16	f = 1.779; P = 0.159 (0.006)
	Left ankle	38.33 ± 3.75	36.98 ± 4.07	38.08 ± 5.22	39.95 ± 4.20	f = 1.406; P = 0.248 (0.005)
	Asymmetry	11.96 ± 5.30	7.06 ± 5.69	5.98 ± 4.37	7.95 ± 3.74	f = 5.510; P = 0.002 (0.186)
**Female**						
	Right ankle	37.95 ± 3.78	34.30 ± 3.15	35.48 ± 3.66	39.02 ± 3.68	f = 6.925; P = 0.001 (0214)
	Left ankle	38.87 ± 3.43	36.32 ± 3.15	37.02 ± 5.71	40.10 ± 3.11	f = 5.373; P = 0.002 (0.174)
	Asymmetry	4.84 ± 4.47	4.20 ± 5.10	5.17 ± 4.50	5.20 ± 5.68	f = 0.585; P = 0.627 (0.002)

* Values are mean ± standard deviation unless otherwise indicated.

**Figures 1 and 2:**
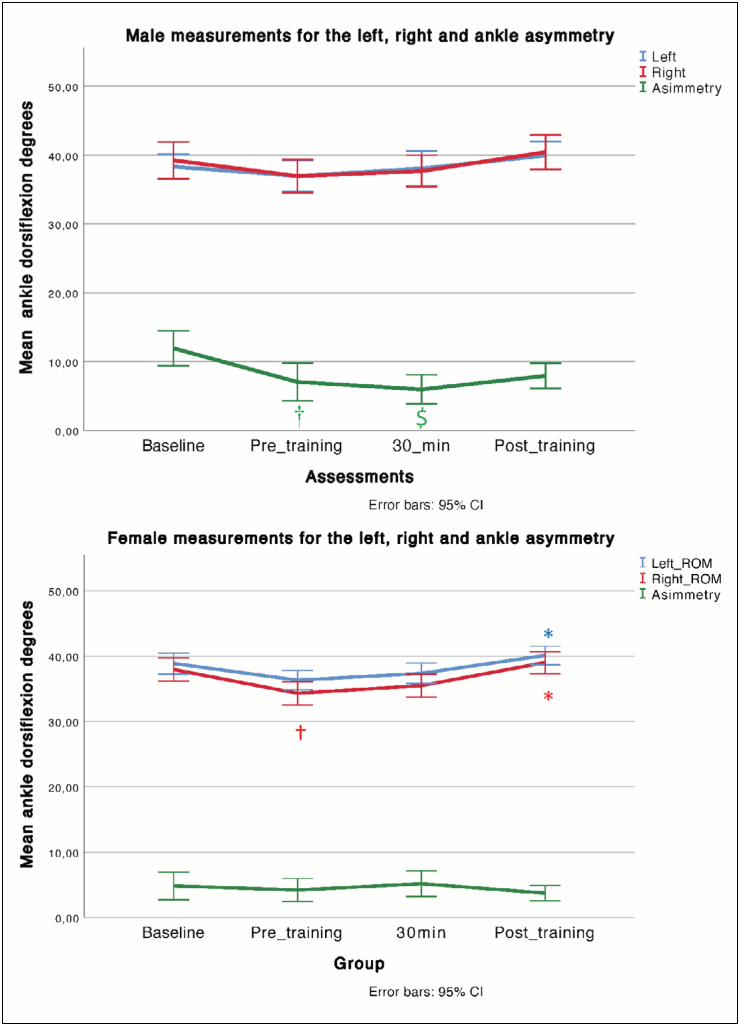
* Significant differences between pre-training and post-training assessments; †, significant differences between the baseline and pre-training assessments; $ significant differences between the baseline and 30-min.

A statistical analysis evaluating the comparison of ankle taping between male and female basketball players did not report significant differences in the time and interaction (time vs. group) for any variable. Bonferroni corrections for the interaction between groups reported differences in the right ankle at baseline, pre-training, pre-training-30-minute training, and pre-training-post-training. For the left ankle, post-training, pre-training 30-minute training, and pre-training-post-training. The asymmetric variable showed significant differences between the baseline and the rest of the variables (Table 3).

**Table 3:** Two-way analysis of variance (ANOVA) and Bonferroni correction values for the intra-subject effects of the total sample

		Two-way ANOVA values		Time versus Group f; P (Eta^2^)
		Time f; P (Eta^2^)		
**Right ankle**		f = 56.809; P = 0.001 (0.606)		f = 1.925; P = 0.130 (0.049)
**Left ankle**		f = 28.318; P = 0.001 (0.434)		f = 1.196; P = 0.315 (0.031)
**Asymmetry**		f = 8.633; P = 0.001 (0.189)		f = 7.899; P = 0.002 (0.176)
**Bonferroni correction values**				
**Measure**		**Right ankle P value**	**Left ankle P value**	**Asymmetry P value**
**Baseline**				
***Pre-training***		0.001	0.001	0.014
	30-minute training	0.001	0.467	0.005
	Post-training	0.009	0.001	0.001
***Pre-training***				
	30-minute training	0.001	0.001	1.000
	Post-training	0.001	0.001	1.000

## DISCUSSION

The present study compared the ankle taping procedure on ankle mobility in four practice moments in which it seeks to achieve an ankle ROM limitation in order to prevent ankle and foot injuries (or re-injuries) in basketball players.^2^ The main findings of the present study suggest that during the first 30 min of practice, ankle taping did not present differences with the baseline in both male and female basketball players. Thus, based on these results, it might be understood that in a typical practice session of 90 min or even in a basketball game of duration of over 120 min, the taping effectiveness represents approximately 25% of the time making this function and is effective in limiting the ankle joint movement. In addition, pro- and semi-professional basketball teams spent approximately 30 to 45 min of warm-up time based on stretching and neuromuscular performance exercises.^22^ These routines were composed of several exercises that involve the ankle joint, such as jumps, calf stretching, or ankle mobility exercises.^23^ Therefore, based on the findings of the present study, the authors suggest that even before the start of a basketball game, ankle taping may decrease the effectiveness of ankle ROM restriction.

Regarding a complete training session or full game, prior research showed that ankle taping decreased the ankle dorsiflexion ROM in U18 basketball players; however, at the end of the training session, the ankle ROM limitation was very low, with the last 30 min of a session or a game being the moment of highest injury (or re-injury) risk in a basketball player.^15^ At the same line, the findings of this study report no differences between the baseline and the end of the training session. In fact, a slight increase in ankle dorsiflexion ROM was observed. These results could be explained by the decrease in ankle taping added to repeatedly performing high-intensity actions, such as jumps, sprinting, change of direction, or landings, which force the ankle joint to maximum dorsiflexion ranges throughout the training session.^24^ Domínguez-Díez et al.^25^ reported that these actions require the implementation of intense accelerations and decelerations, with high impact force peaks, which are directly related to joint overload, increasing the injury risk. Consequently, proper prophylactic approaches are necessary to protect the health and development of players. Several authors have researched other ankle joint restriction approaches as alternatives to rigid tapes, such as semi-rigid bracing methods. For example, Gross et al.^26^ reported that semi-rigid ankle braces are warranted to reduce initial and recurrent ankle sprain injuries in athletes without affecting their functional parameters. Janssen et al. evaluated the effectiveness of combined bracing and neuromuscular training with respect to an isolated bracing approach on the recurrence of ankle sprain injuries in 384 athletes. They reported that the bracing approach was superior to the neuromuscular training in reducing the incidence, but not for the severity of self-reported ankle injury risk.^27^ Authors of the present study argued that these semi-rigid bracing techniques might be more effective throughout time (e.g. a single training session or a game) that a classic taping on the ankle ROM restriction due to the plastic and semi-rigid materials could not be deformed. Regarding the adverse effects on performance, several authors have observed that the type of ankle stabilizer can influence lower limb kinematics, ground reaction forces, and muscular activity contraction. For example, Theodorakos et al.^28^ showed that a semi-rigid ankle brace altered the ankle kinematics owing to the ankle joint ROM restriction. Considering the ground reaction forces, Cordova et al.^20^ showed that external ankle support reduces ankle and knee joint displacement, which influences the space-time features of the ground reaction forces during drop landings.

Regarding the ankle ROM asymmetry, the results of the present study reported asymmetric differences when taping was performed. Moreno-Pérez et al.^29^ showed an increased ankle dorsiflexion ROM after a soccer match for the dominant ankle; however, a decrease at 48 h post-match in both ankles was observed. Moreover, similar values were reported with an increase in the ankle dorsiflexion ROM post-match with respect to the pre-match and a decrease 48 h post-game in both ankles in semi-professional basketball players.^30^ The asymmetry differences in response to the ankle taping procedure could be explained by the restriction of the muscular and ligament structures that surround the ankle joint and by proprioception mechanisms.^31^

Another important aspect to consider was the human resources and taping-time costs, not just for a single practice or game, but during a complete basketball season. A classic taping procedure takes over 3 to 5 min per ankle joint and is approximately three times more expensive than bracing.^32^ However, a semi-rigid bracing ankle approach could be self-dressed barely in 1–2 min without any need for a physiotherapist. Therefore, all these aspects should be considered when planning basketball sessions for medical staff.

A full assessment of ankle and foot structures and features is considered essential for complete athlete exploration, such as the degree of ankle stiffness or structural conditions, such as functional hallux limitus.^33,34^

### Clinical applications

Based on the current literature and findings of the present study, the classic ankle taping method may be useful for decreasing the ankle dorsiflexion ROM in both male and female basketball players to prevent ankle injury. However, the duration of the efficacy was still questioned due to the present results, which did not show differences between the baseline with no taping and at 30 min evaluation and the end of the practice. Therefore, the “dynamic effectiveness” of the classic taping method should be revised to develop new prophylactic approaches, taking into account the ankle ROM restriction effectiveness, human resources, and the time required to develop full ankle taping. For example, the implementation of semi-rigid bracing techniques or the addition of active stripes in the training or game pauses, being the first option the most appropriate assumed strategy.

### Limitations and future studies

This study has a few limitations. First, only one training session was conducted for each group. Second, the height and weight variables were significantly different between the groups.

Additional research is needed to assess the newly available brace approaches and their influence on ankle sprain injury prevention and functional performance. In addition, future studies should compare the effectiveness of classic ankle taping and semi-rigid bracing on ankle sprain injury rates and whether it affects the biomechanics of players. Several authors support ankle bracing as an ankle sprain injury prevention method owing to the restriction of sagittal plane movements. However, future studies are needed to assess whether these prophylactic approaches may have negative effects on the ankle and knee joints. Future research should explore other biomechanical and psychological features, such as asymmetrical values with the idiomatic side or psychological effects of the ankle taping procedure.

## CONCLUSIONS

Ankle taping effectively decreased the ankle dorsiflexion ROM immediately after application in both male and female basketball players. However, ROM restriction was very low after 30 and 90 min, as assessed in a single basketball practice. Therefore, the classic taping method should be revised to develop new prophylactic approaches, such as the implementation of semi-rigid bracing techniques or the addition of active stripes during training or game pauses.
